# Mechanical Property and Structure of Polypropylene/Aluminum Alloy Hybrid Prepared via Ultrasound-Assisted Hot-Pressing Technology

**DOI:** 10.3390/ma13010236

**Published:** 2020-01-06

**Authors:** Kunpeng Du, Jin Huang, Jing Chen, Youbing Li, Chaolong Yang, Xiaochao Xia

**Affiliations:** 1 College of Materials Science and Engineering, Chongqing University of Technology, Chongqing 400054, China; kunpengdu@163.com (K.D.); HJ58824152@163.com (J.H.); 13873300473@163.com (J.C.); yclzjun@163.com (C.Y.); xiaxiaoc@cqut.edu.cn (X.X.); 2 Chongqing Key Laboratory of Mold Technology, Chongqing 400054, China; 3 Key Laboratory of Advanced Manufacturing Technology for Automobile Parts, Ministry of Education, Chongqing 400054, China

**Keywords:** polypropylene/aluminum alloy hybrid, ultrasound-assisted hot-pressing technology, single lap joint, property and structure

## Abstract

The polypropylene/aluminum alloy hybrid was prepared via an ultrasonic-assisted hot-pressing technology (UAHPT). The mechanical property and structure of the UAHPT processed polypropylene/aluminum alloy hybrid were explored by the tensile shear test, scanning electron microscopy (SEM), and atomic force microscopy (AFM), respectively. Prior to obtaining the UAHPT processed hybrid, the microporous structures were prepared by the anodic oxidation in a phosphoric acid solution in which the polypropylene (PP) melt flowed into and formed the micro mechanical interlocking structure at the interface of polypropylene/aluminum alloy. The effects of bonding temperature, pressing pressure, ultrasonic amplitude, and ultrasonic time on the bonding properties of the hybrids were investigated via orthogonal experiment. The UAHPT processed hybrid was strengthened and the maximal tensile shear strength reached up to 22.43 MPa for the polypropylene/aluminum alloy hybrid prepared at the optimum vibration processing parameters.

## 1. Introduction

Recently, the energy crisis is becoming more and more urgent, such as energy consumption and environmental pollution, especially in automobile, electronics, and other industrial fields. The lightweight material application in a car has become one of the most important means of energy-saving and emission reduction technologies, including magnesium alloy [1], aluminum alloy, and polymer. The polymer-metal hybrids (PMH) [2,3] can not only meet the requirements of comprehensive properties of products in the car, but also greatly reduce product weight. Thus, the polymer-metal hybrid technology has been gradually developed in the automobile parts. The PMH processing technologies mainly contain compression molding [4,5,6], injection molding [7,8], and mechanical welding forming [9,10]. Among these technologies, compression molding is an advantageous processing method due to simple and easy control and operation. Arkhurst et al. [4] investigated the effect of resin matrix on the strength of an AZ31 Mg alloy-carbon fiber reinforced plastics (CFRP) joint made by the hot metal pressing technique. This research indicated that the bonding strengths are greatly dependant on the metal oxide layer conducting heat between the metal-plastic materials. Then, they [5] achieved the bonding carbon fiber reinforced thermoplastic polyurethane with AZ31 magnesium alloy through a self-made hot-pressing device, in which feasibility and bonding mechanism was discussed. They found the annealed surface of magnesium alloy has a significant effect on the bonding strength of the joint, whose tensile shear load reaches to ca. 5.1 kN, which is attributed to the annealed oxide layer to inhibit the plastic bubbles. Zhou et al. [6] reported the high modulus carbon fiber reinforced polymer/AZ31 alloy composite was joined by the hot-pressing process, and this study showed that the carbon fiber reinforced polymer/AZ31 composite has higher tensile strength, flexural rigidity, and specific modulus than a traditional magnesium matrix composite. Moreover, the injection molding is another popular PMH technology to produce 3C electronic products. Fabrin et al. [7] studied the effect of microstructure and different etched treatment methods on the metal-plastic adhesion property. They concluded the maximum peel strength of the samples after alkaline-acid pre-treatment reaches 9.33 N/cm. Izadi et al. [8] found the warpage increases with increasing melt temperature for the metal-polymer-metal composites under bending loads. In recent years, mechanical welding as a novel plastic/metal direct adhesion method has gradually become a popular PMH technology. Jiao et al. [9] simulated the PPS-stainless steel laser joining temperature distribution through the finite-element method in the current work, and carried out the laser-based joining experiments, and the results displayed the shear strength reached 17.5 MPa. Liu et al. [10] reported aluminum alloy/polyamide 66 was joined via a friction lap welding and discovered the formation of chemical bonds, which was a key factor for the metal/polymer composites to obtain good bonding strength.

It can be seen from the above literature that the surface pre-treatment for the metal substrate is very important for the preparation of PMH composites, and the surface treatments mainly include coupling agent [11], anodic oxidation [12], chemical etching [13], and laser treatment [14], etc. Honkanen et al. [11] reported the effect of silane treatment on the bonding properties of thermoplastic polyurethane-stainless steel mixtures. The analysis results showed silane layer thickness affects the failure mode of the hybrids, and the hybrids fail mainly in the silane layer with a thinner layer cohesively in plastic. Pan et al. [15] reported the porosity and pore size of the anodic oxide film can be reduced via annealing, which weakened the interface between metal and composite laminates. Yeh et al. [13] discovered the chemical change affects significantly the bonding strength of the hybrids. The bonding strength was improved 2.5 times after the two-step process of chemical etching and plasma treatment. Byskov-Nielsen et al. [14] investigated the mechanical strength of the joint is strongly dependant on the fine laser-generated surface morphology, and the results showed that appropriate surface structuring can increase the bonding strength by several orders of magnitude, realizing a higher mechanical interlocking stability.

Polypropylene (PP) as a general plastic is often used in automobiles, electronics, and other industrial fields. Aluminum alloy is widely used because of its lightweight and good mechanical properties. However, the direct bonding shear strength of polypropylene and aluminum alloy is only 1.6 MPa, which obviously cannot meet the requirements of product service performance [15]. However, in our previous work [16], the bonding properties of aluminum alloy/modified polypropylene (PP) hybrids have been investigated via compression-molded processing technology, and the maximal tensile shear strength of PMH sample has reached 10 MPa. To better improve the bonding strength of the polypropylene/aluminum alloy hybrid sample in the work, ultrasonic-assisted hot-pressing technology (UAHPT) was used to realize the PP and aluminum alloy direction bonding. The processing variables including processing temperature, processing pressure, ultrasonic vibration amplitude, and ultrasonic vibration time were optimized via orthogonal experimental and the failure modes of the polypropylene/aluminum alloy hybrid samples were studied in detail. This provides some reference value for the ultrasonic-assisted pressing process of PP/aluminum alloy.

## 2. Experimental

### 2.1. Materials

Isotactic polypropylene (T30S) was provided by China Petroleum and Natural Gas Co., Ltd. (Beijing, China), whose physical parameters were shown in Table 1. Aluminum alloy (A5754) sheets were provided by Southwest Aluminum Group Co. Ltd. (Chongqing, China), whose main chemical compositions are Al 95.92%, Mg 3.23%, and O 0.87%, and aluminum alloy sheet was cut into dimensions of 25 mm × 100 mm × 2 mm for each experimental sample.

### 2.2. Sample Preparation

The PP granules had been dried at 80 °C for 6 h before injection-molded. A dumbbell plastic sample was injection-molded in the injection machine, and the processing temperatures for the four sections of injection machine were set at 160 °C, 170 °C, 180 °C, and 175 °C in turn. Other processing parameters were displayed in Table 2.

The aluminum alloy sheets were ground with 200#, 400#, 600#, and 800# abrasive papers successively, polished with SiO_2_ polishing paste, and then ultrasonically cleaned with acetone and distilled water. The aluminum alloy surface was treated in order with 5 wt.% sodium hydroxide solution, 10 wt.% nitric acid solution, and deionized water. Then, the treated surface was dried for anodic oxidation. A 10 wt.% of the phosphoric acid solution was selected as an electrolyte, and the electric current was set at 2 A to ensure the current density was 16 A/dm^2^ during anodic oxidation. The oxidation time was set in 15 min and the oxidation temperature was set at 25 °C.

**Figure 1:**
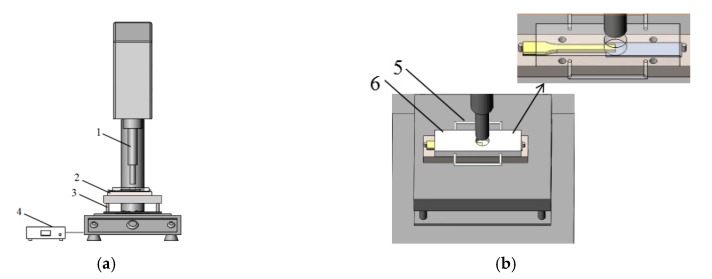
The experimental device for the ultrasonic assisted hot-pressing technology: (**a**) ultrasonic welding machine (1-ultrasound vibration press head, 2-mold, 3-heating table, 4-controller); (**b**) mold diagram (5-adiabatic handle, 6-pressed steel plate).

**Figure 2:**
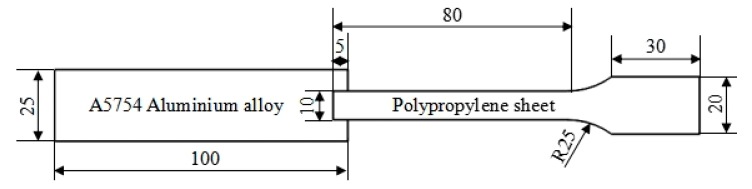
The sketch diagram of single-lap of polypropylene/aluminum alloy hybrid.

The injection-molded polypropylene dumbbell and the surface treated A5754 aluminum alloy sheet were bonded via the self-made, ultrasonic-assisted hot-pressing technology. The UAHPT device was shown in Figure 1, and the PMH sample is also called single-lap specimen, which was shown in Figure 2. The processing equipment contains a split digital indicator to display the ultrasonic-assisted hot-pressing variables. The rated parameters of the ultrasonic generator are: 2200 W rated power, 20 kHz rated frequency, and the amplitude adjustment range is 10–100% (25–48 μm). Firstly, set the ultrasonic time, pressure and amplitude. Then, preheat the A5754 aluminum alloy plate for 15 min at 140 °C. Next, fix the preheat aluminum alloy plate and PP sample in the self-made mold, the overlapping area is 5 mm × 10 mm, and then finally set the temperature to the bonding temperature. When the PP specimen reaches the bonding temperature, the ultrasonic welding joint descends and contacts with the specimen so that the PP/Al alloy begins to bond. When the ultrasonic time is reached, operate the ultrasonic welding joint to rise and wait for the sample to cool before completing the experiment.

### 2.3. Orthogonal Experiment Design

For the ultrasonic-assisted hot-pressing technology, it is very important the effects of bonding temperature, pressing pressure, ultrasonic vibration amplitude, and ultrasonic vibration time on the properties of polypropylene/aluminum alloy hybrids. The above four processing variables were selected as the processing factors during the UAHPT processing, and three levels of each factor were set in the orthogonal experiment design. In other words, the experiments were carried out according to the L_9_(3^4^) orthogonal experimental method. The processing factors and orthogonal experimental design is shown in Table 3.

### 2.4. Tensile Shear Strength Test

All single-lap-joint specimens were tested on a universal testing machine (CMT 6104, Shanghai, China) at a speed of 2 mm/min. To reduce the influence of bending on samples during tension, two gaskets were self-made to clamp UAHPT processed specimens during tension. At the same time, the load-displacement curves were recorded, and the tensile shear strengths were calculated by load-displacement curves.

### 2.5. Surface Morphology Observation

Field emission scanning electron microscopy (FESEM, ZEISS SIGMA, Oberkochen, Germany) was used to investigate the microstructure and topography of the sample surface, including the aluminum alloy surface before bonding and the surface of disrupted specimen after testing. The surface roughness was measured by atomic force microscopy (AFM, Park NX10, Seoul, Korea) in non-contact mode. Furthermore, the tensile fracture morphology was characterized by a tungsten lamp scanning electron microscope (SEM, JSM-6460LV, Tokyo, Japan). The cross-sections of samples were observed to analyze the interface filling between PP and aluminum alloy using FESEM.

## 3. Results and Discussion

### 3.1. Tensile Shear Behavior of the Polypropylene/Aluminum Alloy Hybrid Prepared by UAHPT

Followed by the orthogonal experimental design as shown in Table 3, the PP/aluminum alloy hybrids were individually prepared by ultra-sonic hot-pressing technology. The tensile shear strengths of polypropylene/aluminum alloy hybrids were analyzed by the range analysis method. The results were shown in Table 4 and Table 5, where K1, K2, and K3 are the sum average of the same level, respectively, and R is the range of each factor. Four samples were tested under different process parameters and the average value was taken.

As shown in Table 4, there are significant differences for the tensile shear strength of polypropylene/aluminum alloy hybrids under different forming conditions. The tensile strength ranges from 11.55 MPa to 22.43 MPa. According to the data from Table 5, it can be found that the influence of bonding temperature on the tensile shear strength of specimens is the most significant factor, and ultrasonic vibration amplitude and processing pressure are the secondary factors, while the influence of ultrasonic vibration time on the tensile shear strength of polypropylene/aluminum alloy hybrid the lowest.

**Figure 3:**
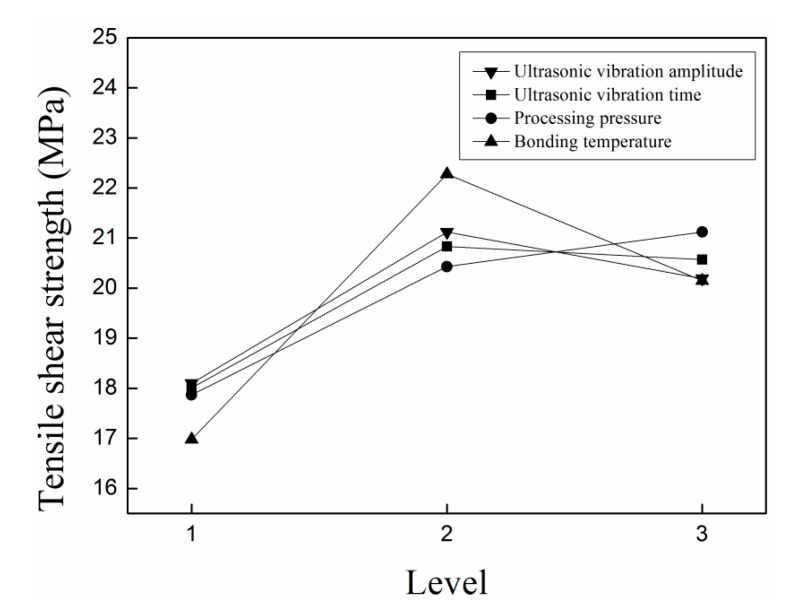
The tensile shear strengths versus UAHPT processing variables.

As shown in Figure 3, the tensile shear strength increases first and then decreases with increasing bonding temperature, ultrasonic vibration amplitude, and ultrasonic vibration time and processing pressure. However, as the bonding temperature, ultrasonic vibration amplitude, and ultrasonic vibration time continue to increase, the excessive fluidity and cavitation of polypropylene occur. Thus, the bond strength between polypropylene and aluminum alloy decreased. After analysis and comparison, the optimum processing parameters of ultrasonic-assisted hot-pressing of polypropylene/aluminum alloy hybrid parts were obtained. The optimum UAHPT parameters are shown in Table 6.

**Figure 4:**
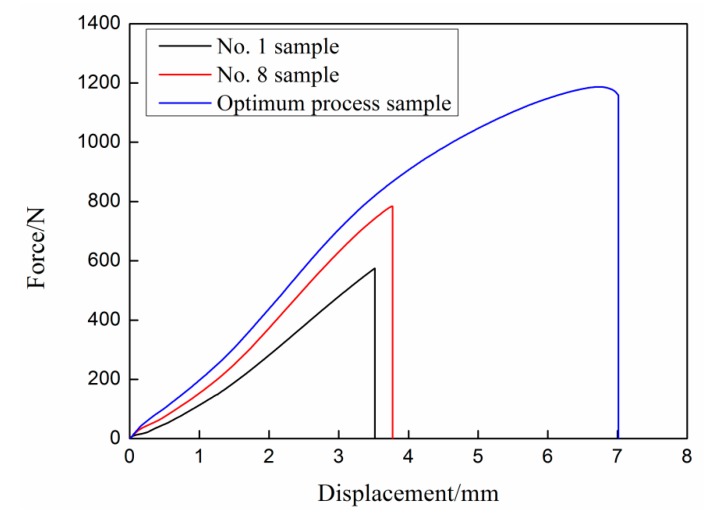
The typical load-displacement curves of the UAHPT prepared specimens.

The tensile shear properties of several typical UAHPT processed samples are described in Figure 4. The displacement and maximum load for the No. 1 specimen are only 3.5 mm and 577 N, respectively, when the sample interface fails. For the No. 8 sample, the failure displacement increases by 0.3 mm and the load increases by ca. 403 N in comparison with No. 1 sample. Applied with the optimum UAHPT processing parameters, the tensile load increases sharply and up to 1159 N. Meanwhile, the failure displacement reaches up to 7 mm. This shows that the bonding properties of polypropylene/aluminum alloy hybrids are effectively improved for applied the ultrasonic-assisted hot-pressing process.

**Figure 5:**
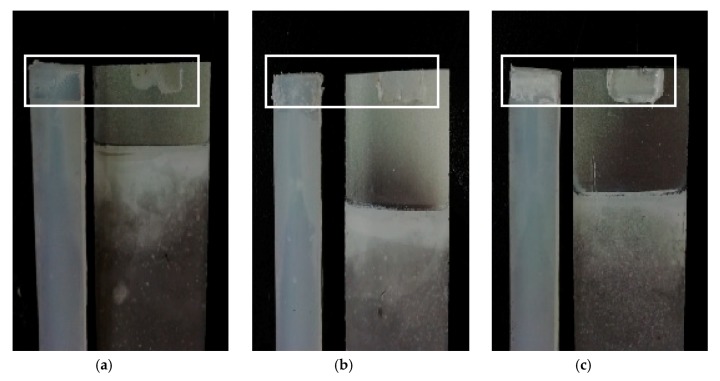
Fracture failure photos of different UAHPT specimens: (**a**) No. 1 sample, (**b**) No. 8 sample, and (**c**) Optimum processed sample.

As seen in Figure 5, it is different that the fracture failure mode of the different specimens after tensile shear fracture failure. Some UAHPT processed single-lap-joint specimens present the mixed failure modes of interface and cohesive failure mode, such as the No. 1 and No. 8 sample failure surfaces, where some resin remains on the aluminum alloy surface. Furthermore, the sample prepared under optimized process conditions fractured in cohesive failure mode during tension as shown in Figure 5c, and many resins remains in the aluminum alloy surface with strong bonding property.

### 3.2. Surface Characterization

**Figure 6:**
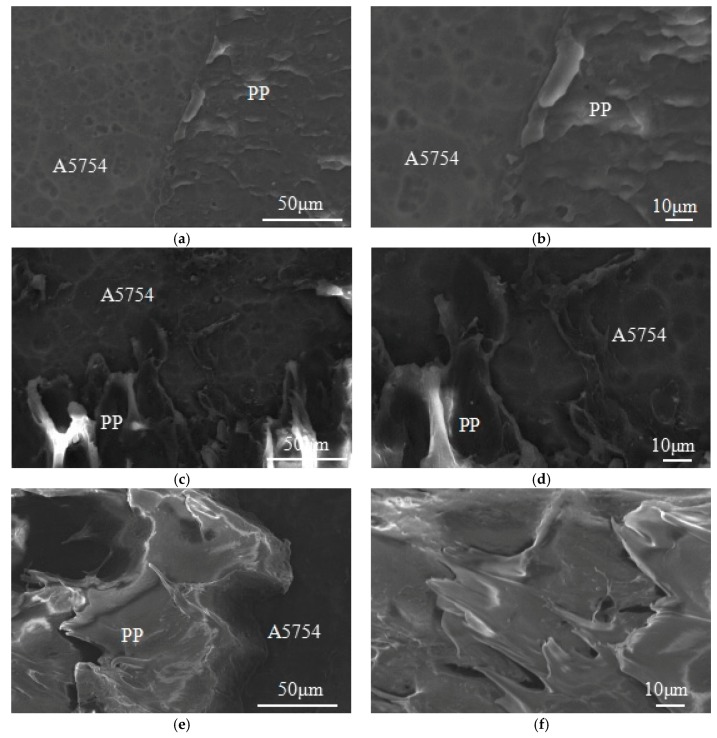
SEM photos of the UAHPT samples after tensile shear test: (**a**) and (**b**) NO.1sample, (**c**) and (**d**) NO.8 sample, (**e**) and (**f**) Optimum processed sample.

To further observe microscopic fractograph of the single-lap specimens after tensile shear fracture failure, the SEM photos of the fracture failure on the aluminum alloy side are shown in Figure 6. As shown in Figure 6a, many anodized holes existing on the aluminum alloy surface. At a higher magnification as shown in Figure 6b, the most region of aluminum alloy surface is relatively smooth and there are less resin residues on aluminum alloy surface with the corresponding relatively weak bonding properties for the No. 1 sample. Compared with processing conditions of No. 1 sample, No. 8 sample was prepared at relatively longer vibration time, relatively higher vibration amplitude, and melt temperature. Thus, this resulted in relatively higher tensile shear strength and bigger elongation at break. After tensile shear fracture failure, there are a lot of PP resin remains on the aluminum alloy surface as shown in Figure 6c,d. The trend to increase plastic deformation of PP resin is good proof of better adhesion of No. 8 sample than that of No. 1 sample. For the optimum processed sample as showed in Figure 6e,f, a large number of PP resins occupied most of the aluminum alloy surface and presented plastic deformation, which is corresponding with the tensile shear behavior for the optimum processed sample. The optimum processed sample was prepared at a relatively longer vibration time and a higher processing pressure against the processing conditions of No. 8 sample, which lead to much higher shear tensile strength than the other samples. It is also proved that the PP melt easy filling micropores on the aluminum alloys surface to form micro-interlocking structures at the interface with strongly bonding properties of the UAHPT processed PP/aluminum alloy hybrids.

**Figure 7:**
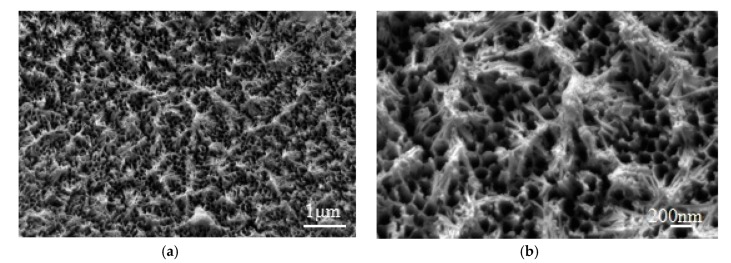
The microstructures of the anodized aluminum alloys surface: (**a**) ×10,000, (**b**) ×30,000.

The fracture behavior is closely related to metal surface morphology. The aluminum alloy surface was treated by phosphoric acid anodization, and the corresponding surface morphology is shown in Figure 7a,b. It is intuitive to see that there are many needle honeycomb-like holes on the anodized aluminum alloy surface, where the micropore size ranges from 150 to 200 nm. When the microstructures are magnified, it is found that the micropores are very close together and some holes run through each other. During the UAHPT process, under the combined effects of ultrasonic vibration and processing pressure, the plastic melts tend to flow into the needle-shaped honeycomb-like microstructure holes, forming the micro-rivet structures to strengthen the adhesion of the aluminum alloy and polypropylene after the polypropylene solidifies.

**Figure 8:**
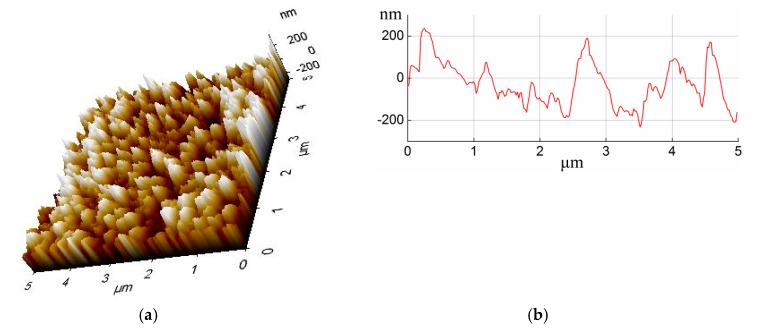
The AFM topography of the anodized aluminum alloy surface: (**a**) atomic force morphology, (**b**) hole depth.

Furthermore, it has already been proven that the relatively rough aluminum alloy surface helps improve the bonding strength of aluminum alloy and polypropylene hybrids in our previous work [17], and the anodic oxidation was used to prepare rough aluminum alloy surface. The surface structure of aluminum alloy was observed through AFM, and four geometric parameters of the film surface structure were obtained including high-low difference Rpv, the root-mean-square roughness Rq, average roughness Ra, average height Rz The AFM topography on the anodized aluminum alloy surface is displayed in Figure 8, which was probed in an overlapping area of 5 μm × 5 μm. The AFM parameters for the anodized aluminum alloy surface were listed in Table 7. The AFM topography of the anodized aluminum alloy surface is consistent with the SEM results. The needle-like microstructure takes on different heights. The maximal depth of the microstructure is up to ca. 220 nm, as shown in Figure 8b, and the mean surface roughness (Ra) are about 89.95 nm, as listed in Table 7. The relatively rough aluminum alloy surface is beneficial to increase the bonding strength for the UAHPT processed metal-plastic hybrid.

### 3.3. Microstructure Characteristics at Joint Interface

**Figure 9:**
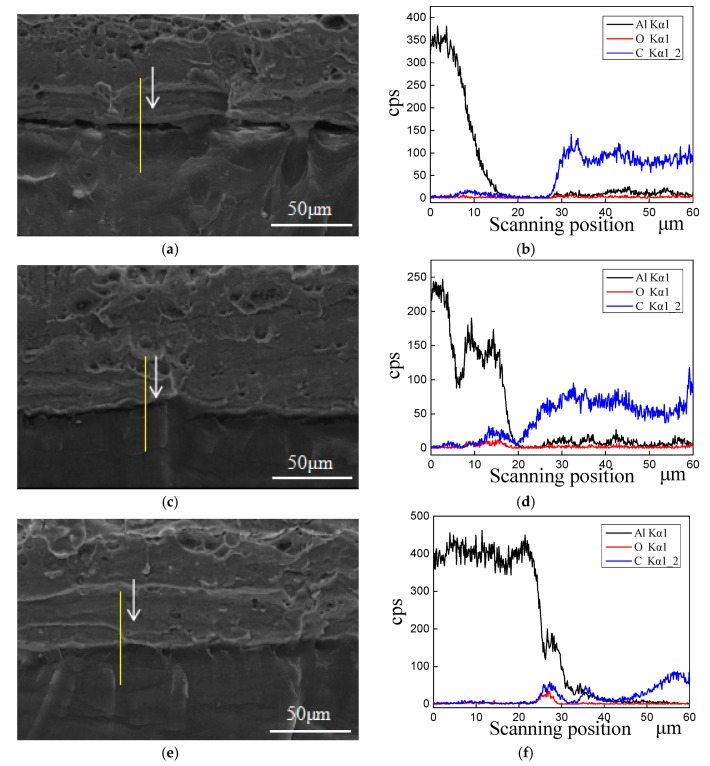
The SEM photos of the cross sections of UAHPT samples: (**a**) No.1, (**c**) No.8, (**e**) Optimum processed sample; the line scan chemical analysis at the interfaces of samples: (**b**) No.1, (**d**) No.8, and (**f**) Optimum processed sample.

To understand the binding mechanism at the joint interface of polypropylene and aluminum alloy, the SEM with line scan chemical analysis was applied to investigate the microstructures and atomic diffusion at the interface. The micrographs of cross-section and atomic migration at the interface for the single-lap samples prepared by different vibration conditions were shown in Figure 9. Among them, the transverse coordinate is the scanning position of the sample, and the longitudinal coordinate represents the characteristic X-ray counting intensity of the element are shown in Figure 9b,d,f. The content of the element can be determined qualitatively by the trend of cps. As shown in Figure 9a, there are relatively obvious gaps at the joint interface in the cross-section view for the No. 1 sample, which indicates that there are few micro-interlocking structures at the interface of PP and aluminum alloy during the UAHPT process. Processed under the optimized UAHPT processing parameters, the gaps at the interface for No. 8 sample reduced as displayed in Figure 9c, and the PP resin and aluminum alloy are tightly closed at the interface for the optimized processed sample, as shown in Figure 9e. The tightly close interface or the optimized processed sample means most of PP resin flowed into the micro-holes on the Al surface with strong adhesion than that of No. 1 and No. 8 samples. It can be concluded that the strong joint strength for the optimum processed specimen is mainly attributed to many PP resin flowing into the needle-shaped, honeycomb-like pores, and forming interlocking microstructures.

The diffusions of aluminum and carbon atoms at the interface of PP and Al are confirmed by line scan chemical analysis, which may justify chemical reactions at the interface for the UAHPT processed samples. As shown in Figure 9b, the C and Al atom migration hardly happens at the interface, thus the joint interface of No.1 sample mainly depends on Van Der Waals force, which is the reason for the poor bonding properties in the No.1 sample. As shown in Figure 9d,f, there is atomic migration at the interfaces for No.8 and the optimum processed sample which supported the increased bonding properties. This is in line with the former researcher’s reports [18,19]. Kimiaki et al. [18] used friction lap welding to bond carbon fiber reinforced PA6 directly to aluminum alloy treated by wet grinding, and the results showed that the -NH and -CH functional groups in PA6 formed chemical bonds with metal surface oxides and Al(OH)_3_, resulting in a maximum tensile strength. Chen et al. [19] discussed that the effect of maleic anhydride grafting amount in polypropylene on lap shear strength of aluminum/PP/aluminum sheet after surface pretreatment with sandpaper grinding aluminum sheet. It showed that the chemical interactions between -OH, Al^3+^, or amino group -NH_2_ at the surface of the aluminum sheets and the polar functional anhydride groups and carboxylic groups -COOH on PP-g-MAH at the interface, which results in fracture occurs in the polymer and shows severe plastic deformation.

## 4. Conclusions

The polypropylene/aluminum alloy hybrids with strong tensile shear strength were prepared via ultrasonic-assisted hot-pressing technology (UAHPT) in the work, and the maximal tensile shear strength reached up to 22.43 MPa for the hybrid processed at the optimum vibration processing parameters. Furtherly, through the orthogonal experimental design, the optimum processing parameters to prepare strong UAHPT processed polypropylene/aluminum alloy hybrid were as follows: ultrasonic vibration amplitude was 50 %, ultrasonic vibration time was 5 s, pressing pressure was 0.6 MPa, pressing temperature was 175 ℃, and pressing time was 15 s. With the application of the optimum UAHPT processing parameters, a large amount of the PP resin flowed into the needle-shaped, honeycomb-like pores on the anodized aluminum alloy surface, forming many micro interlocking structures with corresponding improved bonding properties. The results are of great significance to the optimization of the preparation process of polypropylene/aluminum alloy hybrids. However, further research and improvement are needed to extend the results to another polymer/metal forming.

## Figures and Tables

**Table 1:** The physical properties of polypropylene.

Density (g/cm^3^)	Melt Index (g/10 min)	Vicat Softening Point (°C)	Shrinking Percentage (%)	Tensile Strength (MPa)
0.91	13	120	1.5–20	33

**Table 2:** The processing parameters of the injection-molding.

Injection Pressure (MPa)	Holding Pressure (MPa)	Holding Time (s)	Cooling Time (s)	Screw Speed (rpm)
40	30	6	20	40

**Table 3:** The processing factors and orthogonal experimental design.

Level	Factors			
	Ultrasonic Vibration Amplitude (%)	Ultrasonic Vibration Time (s)	Processing Pressure (MPa)	Bonding Temperature (°C)
1	10	2	0.1	165
2	10	5	0.3	175
3	10	10	0.6	185
4	50	2	0.3	185
5	50	5	0.6	165
6	50	10	0.1	175
7	100	2	0.6	175
8	100	5	0.1	185
9	100	10	0.3	165

**Table 4:** Tensile shear strengths of the UAHPT processed samples followed by the orthogonal experimental design.

Specimens	Tensile Shear Strength (MPa)
1	11.55
2	22.23
3	20.53
4	20.29
5	20.64
6	22.43
7	22.18
8	19.62
9	18.76

**Table 5:** The range analysis of tensile shear strengths of the UAHPT processed samples.

Level	Factors			
	Ultrasonic Vibration Amplitude (%)	Ultrasonic Vibration Time (s)	Processing Pressure (MPa)	Bonding Temperature (°C)
K1	18.10	18.01	17.87	16.98
K2	21.12	20.83	20.43	22.28
K3	20.19	20.57	21.12	20.15
R	3.02	2.82	3.25	5.30

**Table 6:** The optimum processing parameters of UAHPT.

Ultrasonic Vibration Amplitude (%)	Ultrasonic Vibration Time (s)	Processing Pressure (MPa)	Bonding Temperature (°C)	Hot-Pressing Time (s)
50	5	0.6	175	15

**Table 7:** The AFM parameters for the anodized aluminum alloy surface.

Aluminum Alloy Sample	Rpv (nm)	Rq (nm)	Ra (nm)	Rz (nm)
	445.94 ± 0.11	107.76 ± 0.24	89.95 ± 0.16	388.94 ± 0.42
